# A web-based normative calculator for the uniform data set (UDS) neuropsychological test battery

**DOI:** 10.1186/alzrt94

**Published:** 2011-11-11

**Authors:** Steven D Shirk, Meghan B Mitchell, Lynn W Shaughnessy, Janet C Sherman, Joseph J Locascio, Sandra Weintraub, Alireza Atri

**Affiliations:** 1 Department of Neurology, Massachusetts General Hospital Memory Disorders Unit, 15 Parkman Street, WACC 715, Boston, MA 02114, USA; 2 Harvard Medical School, 25 Shattuck Street, Boston, MA 02115, USA; 3 Geriatric Research Education and Clinical Center, Edith Nourse Rogers Memorial Veterans Hospital, 200 Springs Road, Bedford, MA 01730, USA; 4 Department of Psychiatry, Massachusetts Mental Health Center, 180 Morton Street, Jamaica Plain, MA 02130, USA; 5 Department of Psychiatry, Beth Israel Deaconess Medical Center, 330 Brookline Avenue, Boston, MA 02215, USA; 6 Cognitive Neurology and Alzheimer's Disease Center, Northwestern University Feinberg School of Medicine, 320 E. Superior, Searle 11-467, Chicago, IL 60611, USA

**Keywords:** Alzheimer's disease, cognitive aging, MCI, memory, norms

## Abstract

**Introduction:**

With the recent publication of new criteria for the diagnosis of preclinical Alzheimer's disease (AD), there is a need for neuropsychological tools that take premorbid functioning into account in order to detect subtle cognitive decline. Using demographic adjustments is one method for increasing the sensitivity of commonly used measures. We sought to provide a useful online *z-score *calculator that yields estimates of percentile ranges and adjusts individual performance based on sex, age and/or education for each of the neuropsychological tests of the National Alzheimer's Coordinating Center Uniform Data Set (NACC, UDS). In addition, we aimed to provide an easily accessible method of creating norms for other clinical researchers for their own, unique data sets.

**Methods:**

Data from 3,268 clinically cognitively-normal older UDS subjects from a cohort reported by Weintraub and colleagues (2009) were included. For all neuropsychological tests, *z-scores *were estimated by subtracting the raw score from the predicted mean and then dividing this difference score by the root mean squared error term (RMSE) for a given linear regression model.

**Results:**

For each neuropsychological test, an estimated *z-score *was calculated for any raw score based on five different models that adjust for the demographic predictors of SEX, AGE and EDUCATION, either concurrently, individually or without covariates. The interactive online calculator allows the entry of a raw score and provides five corresponding estimated *z-scores *based on predictions from each corresponding linear regression model. The calculator produces percentile ranks and graphical output.

**Conclusions:**

An interactive, regression-based, normative score online calculator was created to serve as an additional resource for UDS clinical researchers, especially in guiding interpretation of individual performances that appear to fall in borderline realms and may be of particular utility for operationalizing subtle cognitive impairment present according to the newly proposed criteria for Stage 3 preclinical Alzheimer's disease.

## Introduction

The Uniform Data Set (UDS) neuropsychological test battery is administered to research participants at all contributing Alzheimer's Disease Centers (ADCs) and Alzheimer's Disease Research Centers (ADRCs) [1]. However, because the subjects are not reflective of the national population and the tests within the UDS battery were modified for pragmatic use, reliable normative data are not available for the battery. Weintraub and colleagues [2] provided descriptive information from initial neuropsychological data of over 3,000 clinically cognitively normal, older adults and developed linear regression models to estimate the impact of age, sex, and education on test performance. The report by Weintraub *et al*. provided in-depth descriptive information about cognitively normal older adults in the UDS, but was not intended as a normative study. By combining the initial results of Weintraub and colleagues with additional statistical information obtained from the study's authors (for example, root mean square errors for model variables), we sought to create a useful regression-based norms calculator that provides estimated *z-scores *while taking into consideration the individual's sex, education level, and/or age and to make this straightforward tool available on the web for clinical research use at the National Institute on Aging Alzheimer's Disease program UDS sites. In addition, we aimed to provide an easy and accessible method for calculating norms which other researchers and clinicians can apply to their own unique, site-specific data sets.

With the recent publication of revised diagnostic criteria for the Alzheimer's disease spectrum by the National Institute on Aging and Alzheimer's Association work-groups (NIA-AA) [3], there is an increased appreciation of detecting subtle cognitive decline in its preclinical stage. Sperling and colleagues propose three stages of preclinical AD, beginning decades prior to clinical symptoms with stage 1, characterized by asymptomatic amyloid deposition in the brain; stage 2, characterized by continued amyloid deposition and the beginnings of neurodegeneration; and stage 3, characterized by continued progression of amyloid deposition, neurodegeneration and very subtle cognitive impairments. These three stages are proposed to precede the stage of mild cognitive impairment (MCI), and as such, the subtle cognitive decline in stage 3 is, by definition, difficult to detect with many neuropsychological tests without consideration of a premorbid level of functioning [3]. In the absence of neuropsychological test data on an individual's level of cognitive functioning prior to disease onset, as is often the case in clinical research settings, the use of demographically adjusted norms can be used to improve the sensitivity of traditional measures.

## Materials and methods

### Subjects

Data used for this study were those from older adult subjects included in the Weintraub *et al*. [2] report. The subjects were deemed clinically cognitively-normal during an initial UDS assessment on the basis of the following criteria: 1) a Clinical Dementia Rating (CDR) [4] Global score of 0; 2) a Functional Assessment Questionnaire (FAQ) [5] score of 0; 3) no other indications of cognitive decline or dementia based on information from supplemental questionnaires; and 4) having a complete set of data including demographics, such as age, education and sex. From an initial data set of 11,287 subjects, 3,268 met the above criteria. Of those 3,268 subjects, 65.8% were female, 81.8% were White, 12.8% were Black, 4.2% were Hispanic, and 1.2% identified as Non-Hispanic Other. The age breakdown for subjects was as follows: 8.6% < 60, 25.6% between 60 and 69, 39.9% between 70 and 79, 22.2% between 80 and 89, and 3.7% ≥ 90 years old. The education profile (years of education) for subjects was as follows: 20.4% ≤ 12 years, 21.0% between 13 and 15 years, 24.0% with 16 years, and 34.7% ≥ 17 years of education.

### Neuropsychological tests

We included the following UDS neuropsychological tests: Mini-Mental State Examination (MMSE) [6], Wechsler Memory Scale-Revised (WMS-R) subtests Logical Memory IA and IIA [7], Digit Span Forward and Backward [7], Semantic Fluency (Animals and Vegetables) [8], Boston Naming Test (BNT) (30 item - odd numbered) [9], Wechsler Adult Intelligence Scale-Revised (WAIS-R) Digit Symbol Coding subtest [10], and Trail Making Test (TMT) Parts A and B [11]. Detailed discussion of the modifications made to these measures for the purpose of the UDS is found in Weintraub *et al*. [2].

### Calculation of *z-scores *

We used the following equation to calculate *z-scores *in our models:

(1) $$Z=\frac{Y-\bar{Y^{\prime }}}{RMSE}$$

where:

*Z *is the *z-score *estimate for an individual subject

*Y *is the raw score for an individual subject obtained from performance on a given test

${\bar{Y}}^{\prime }$ is the predicted population mean score and,

*RMSE *is the root mean square error of the regression equation, which we substitute as an estimate for a population standard deviation (see below).

For each neuropsychological test (NPT), and using Equation 1, we created modified simple regression equations that are conditioned on a single demographic variable (Univariate Models (UV)), as well as a multiple regression equation specific to a set of demographic variables (Multivariate Model (MV)). Because a lower score on TMT A and B is indicative of better performance, the *z-score *estimates for these two measures were reversed. We used regression coefficients from Table 5 of Weintraub *et al*. [2] to first predict, using the MV model (SEX, AGE, and EDUCATION combined), the mean of the theoretical population for an individual subject with the same age (years), education (years), and sex (coded as 1 = male, 2 = female). We then repeated this process using a regression coefficient obtained from a UV model (SEX, AGE, or EDUCATION). Finally, we calculated a *z-score *estimate without any consideration of sex, age or education (Unconditional model, (UC)).

## Results

Table 5 in Weintraub *et al*. [2] shows the coefficients for the variables in the multivariate regression model for estimating the MMSE as a function of SEX, AGE, and EDUCATION (MV model), and we can write the corresponding regression equation as:

(2) $${\bar{Y^{\prime }}}_{\text{MMSE}}=28.41+0.48*\text{SEX + - 0}\text{.02*AGE + 0}\text{.14*EDUCATION}$$

For illustrative purposes, if we are interested in predicting the mean MMSE for a theoretical population of 80-year-old men with 12 years of education, we enter these variables into Equation 2 (that is, SEX = 1, AGE = 80, EDUCATION = 12) to obtain a predicted MMSE mean of 28.04 (that is,${\bar{Y^{\prime }}}_{\text{MMSE}}=\text{MMSE}$ (1, 80, 12) = 28.04). Next, if we would like to obtain an estimated *z-score *(and ultimately percentile rank) for the MMSE score of a particular 80-year-old man with 12 years of education who scores a 27 on the MMSE, then we must first subtract the predicted mean (${\bar{Y^{\prime }}}_{\text{MMSE}}$) 28.04 from the subject's score (*Y*_MMSE_) of 27. Then, we need to divide this difference (that is, $Y_{\text{MMSE}}-{Ȳ}_{\text{MMSE}}^{\prime }=27-28.04$) of -1.04 by the standard deviation of the theoretical population. Pragmatically, we are unable to calculate the standard deviations for all possible combinations of age, education, and sex by which to divide the difference between the subjects' test scores and the predicted mean. Since we are limited to the use of only the information available from Weintraub *et al*. [2], along with the corresponding root mean square errors (RMSE), we are unable to calculate predicted standard deviations for each age, education, and sex combination without the raw data for all subjects. Therefore, we instead substitute the RMSE of each regression equation as an estimate of the standard deviation.

The RMSE is the square root of the average squared differences between the observed score and the predicted score, which gives us an approximation of the average deviation around each of the predicted means for each model. The formula for calculating the RMSE is:

(3) $$RMSE=\sqrt{\frac{{\sum \left(Y-Y^{\prime }\right)}^{2}}{n-k-1}}$$

where:

*RMSE *is the root mean squared error,

*Y *is the observed NPT score,

*Y*' is the predicted NPT score,

*n *is the number of observations and,

*k *is the number of predictors/covariates.

**Figure 1:**
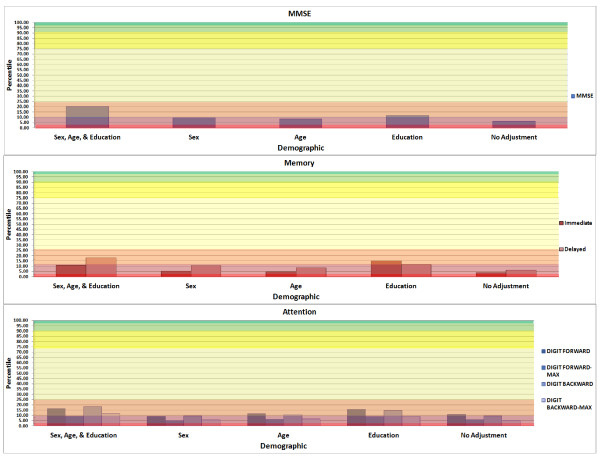
**Examples of graphical output provided by online calculator for MMSE, memory and attention**. MMSE, Mini-Mental State Examination

**Figure 2:**
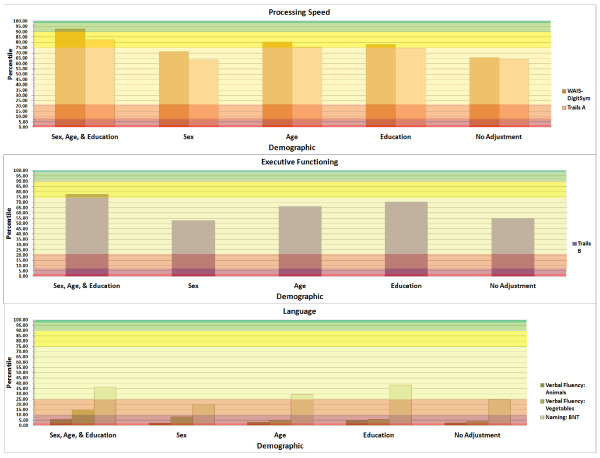
**Examples of graphical output provided by online calculator for processing speed, executive functioning and language**. BNT, Boston Naming Test; TMT, Trail Making Test; WAIS DigitSym, Wechsler Adult Intelligence Scale Digit Symbol Coding.

Most statistical packages include the RMSE in the output (for example, Statistical Analysis Software (SAS), Statistical Package for the Social Sciences (SPSS), STATA and M*plus*), but it may be labeled differently (for example, SPSS labels it the standard error of the estimate). For the above example, the RMSE is 1.24; therefore, we can estimate the subject's *z-score *as -1.04/1.24 = -0.84. The value corresponds to a percentile score of 20.14, and we have thus obtained one estimate, using the MV model, of the subject's performance on the MMSE as approximately at the 20^th ^percentile. Repeating this process using the different RMSEs for each of the UV models for SEX, AGE, and EDUCATION, and the UC model, provides different *z-scores *and percentile estimates of 9.49, 8.41, 11.88, and 6.20 percentiles, respectively. Table 1 depicts output from the online calculator. Figures 1 and 2 provide an example of the graphical representation of the results for this particular example.

**Table 1:** Example Output from the UDS Online Calculator

Sex (M = 1; F = 2)	1										
Age	80										
Education	12	Sex, age, & education		Sex only		Age only		Education only		No adjustment	
		***Z *Score**	**Percentile**	***Z *Score**	**Percentile**	***Z *Score**	**Percentile**	***Z *Score**	**Percentile**	***Z *Score**	**Percentile**
MMSE	27	-0.837	20.14	-1.306	9.59	-1.378	8.41	-1.181	11.88	-1.538	6.20
Logical Memory A: Immediate	7	-1.242	10.71	-1.614	5.33	-1.706	4.40	-1.033	15.09	-1.769	3.84
Logical Memory A: Delayed	6	-0.922	17.84	-1.261	10.37	-1.388	8.26	-1.190	11.71	-1.535	6.24
Length of Delay (25-35 min)	35										
Digit Span Forward	6	-0.980	16.36	-1.333	9.12	-1.192	11.66	-1.004	15.77	-1.238	10.78
Digit Span Forward: Length	5	-1.341	9.00	-1.642	5.03	-1.518	6.45	-1.347	8.90	-1.545	6.11
Digit Span Backward	4	-0.910	18.15	-1.308	9.54	-1.252	10.52	-1.044	14.82	-1.318	9.37
Digit Span Backward: Length	3	-1.207	11.38	-1.576	5.75	-1.514	6.50	-1.326	9.24	-1.667	4.78
Category Fluency: Animals	9	-1.568	5.85	-1.989	2.33	-1.836	3.32	-1.667	4.77	-1.964	2.47
Category Fluency: Vegetables	7	-1.037	14.99	-1.391	8.20	-1.628	5.17	-1.578	5.73	-1.750	4.01
Trails A	29	0.934	82.48	0.349	63.66	0.689	75.46	0.653	74.31	0.364	64.19
Trails B	84	0.759	77.59	0.065	52.60	0.413	66.02	0.533	70.28	0.126	55.01
WAIS-Digit Symbol	52	1.441	92.52	0.559	71.19	0.854	80.33	0.772	77.98	0.400	65.54
Boston Naming Test (30 odd)	25	-0.346	36.46	-0.843	19.96	-0.551	29.09	-0.294	38.43	-0.688	24.59

MMSE, Mini-Mental State Examination; WAIS, Wechsler Adult Intelligence Scale.

Severely impaired = < 1%, Moderately impaired = 1% to 1.99%, Mildly impaired = 2% to 8.99%, Low average = 9% to 24.99%, Average = 25% to 74.99%, High average = 75% to 90.99, Superior = 91% to 97.99%, Very superior = 98%<.

For the neuropsychological tests, we created a table that provides estimated *z-scores *for each model (MV model, UV models, UC model) corresponding to the demographic predictor variables (that is, the SEX, AGE, EDUCATION) concurrently, individually, or without consideration of any of these covariates. To facilitate efficiency, accuracy and utility, we developed an interactive online calculator that allows the entry of a raw score on any UDS neuropsychological test and provides five corresponding estimated *z-scores *based on predictions from each corresponding model. The calculator also produces a corresponding percentile rank and its graphical representation. The full interactive calculator with the above example is provided in Additional file 1 as well as our website [12]. The calculator was created on a Windows^® ^OS using Microsoft Office 2007^® ^and requires Microsoft Office 2007 (Microsoft Corporation, Redmond, WA, USA) for full functionality.

## Discussion

This paper presents a simple method that builds on models reported by Weintraub and colleagues [2] to create a calculator that can provide NACC and ADC clinical researchers with a quick, efficient, and straightforward means to obtain a range of *z-scores *and percentile rank estimates for performance of subjects on the neuropsychological tests of the UDS. In addition, the method we present in this paper can be easily modified so that other researchers and clinicians may conduct their own linear regressions, obtain the necessary output, and create their own norms calculator for their specific site. Furthermore, in the absence of their own available data, researchers can apply this technique to other published data to derive demographically specific norms for a given sample. A generic calculator has been provided in the supplemental materials, which can be used as a template (Additional file 2).

We estimated a range of *z-scores *for individual performance on UDS neuropsychological tests by utilizing coefficients (βs) for demographic variables (predictors) for multivariate (MV) and univariate (UV) linear regression models provided by Weintraub and colleagues [2], as well as corresponding model RMSE terms for test scores of over 3,000 clinically cognitively normal subjects. In employing the RMSE, we leveraged two assumptions that are presumed when testing the significance of predictors in a regression: 1) that the distribution of the residuals around the estimate is normal and 2) that the distribution of the residuals is homoscedastic. The RMSE is an approximately unbiased estimate of the standard distribution of the residuals and, therefore, may provide a reasonable estimate of the distribution across changes in the predictor variable. For example, if one were to perform a simple linear regression and use age as the sole predictor for the MMSE score, one would assume that the error between the predicted MMSE scores and the actual MMSE scores are the same across different ages. This estimate in turn provides one with a measure of the average deviation for any age, and can be substituted for the conventional standard deviation. This approach can then be expanded to any simple or multiple regression model to provide an estimate of the standard deviation of various theoretical population means.

A point of long debate during local ADC/ADRC UDS consensus conferences is whether an individual who performs in the below average range on one or more neuropsychological tests or the MMSE has performed in the impaired range or low-average range. Since the battery contains slight variations in administration procedures, modifications to some of the original measures, and the subjects are not reflective of the national population [2], most norms available for wide clinical use do not apply, leaving UDS researchers with few practical resources to assess performance of subjects on neuropsychological domains other than summary data and models from Weintraub *et al*. [2] and local or national summary statistics that function much like the unconditional (UC) model shown here [13]. However, our example highlights an important point for subjects whose performance falls near the peripheries. The hypothetical subject's performance of 27 on the MMSE is estimated at the sixth percentile relative to clinically cognitively-normal subjects in the UDS, without considering the individual's sex, age, or education (that is, the UC model); it is greater than -1.5 SD and would be perceived to be in the mildly impaired range. However, if other models are used that take into consideration the individual's sex, age, and/or education, his performance is then estimated as at the 8^th ^percentile with sex-conditional (UV_SEX _model), 10^th ^percentile with age-conditional (UV_AGE _model), 11^th ^percentile with education-conditional (UV_EDUCATION _model), or as high as 20^th ^percentile with all covariates considered (that is, the MV model). In this specific example, considering any demographic variable, (sex, age, or education), results in a change in perception of the subject's performance from being greater than 1.5 SD below the mean to falling in the range of -1.5 to 1.0 SDs. Finally, considering all demographic covariates in the MV model results in a finding that the subject has not performed in the mildly impaired range but in the low-average range of -1.0 to -0.5 SDs. The variation in clinical classification, based on which normative considerations are made, becomes even more relevant to MCI and AD diagnosis when considering performance on memory-specific measures. If, for example, a 60-year-old male subject, who is highly educated (for example, 20 years of education), recalled four story units on delayed recall after a 25-minute delay (that is, LMIIA = 4; Table 2), performance estimates range from the 2^nd ^percentile in the UC model, representing mildly impaired performance, to estimates ranging from the < 1 to 3.4^th ^percentiles for the UV models, and an estimated performance at the 0.8^th ^percentile for the MV model, representing performance in the severely impaired range. Such differences may have important implications for cross-sectional and longitudinal classifications that are made on the basis of percentile or categorical thresholds, such as sufficiently impaired performance to meet MCI or AD criteria. In addition, use of the same model for determining performance on measures is critical for accurately modeling and assessing a patient's functioning across time (that is, to determine progression of cognitive functioning).

**Table 2:** An example where performance falls in normal or impaired range depending on demographic adjustment/model used

Sex (M = 1; F = 2)	1										
Age	60	Sex, age, & education		Sex only		Age only		Education only		No adjustment	
Education	20										
		***Z *Score**	**Percentile**	***Z *Score**	**Percentile**	***Z *Score**	**Percentile**	***Z *Score**	**Percentile**	***Z *Score**	**Percentile**
MMSE	27	-2.087	1.84	-1.306	9.59	-1.758	3.94	-2.052	2.01	-1.538	6.20
Logical Memory A: Immediate	7	-2.162	1.53	-1.614	5.33	-1.887	2.96	-1.033	15.09	-1.769	3.84
Logical Memory A: Delayed	4	-2.404	0.81	-1.826	3.39	-2.116	1.72	-2.481	0.65	-2.000	2.28
Length of Delay (25-35 min)	25										
Digit Span Forward	6	-1.860	3.14	-1.333	9.12	-1.432	7.61	-1.706	4.40	-1.238	10.78
Digit Span Forward: Length	5	-2.089	1.84	-1.642	5.03	-1.701	4.44	-1.966	2.46	-1.545	6.11
Digit Span Backward	4	-1.910	2.81	-1.308	9.54	-1.511	6.54	-1.834	3.33	-1.318	9.37
Digit Span Backward: Length	3	-2.160	1.54	-1.576	5.75	-1.772	3.82	-2.072	1.91	-1.667	4.78
Category Fluency: Animals	9	-2.928	0.17	-1.989	2.33	-2.357	0.92	-2.565	0.52	-1.964	2.47
Category Fluency: Vegetables	7	-2.051	2.01	-1.391	8.20	-2.119	1.71	-2.031	2.11	-1.750	4.01
Trails A	29	-0.461	32.25	0.349	63.66	-0.131	44.78	0.017	50.67	0.364	64.19
Trails B	84	-0.887	18.76	0.065	52.60	-0.360	35.93	-0.380	35.21	0.126	55.01
WAIS-Digit Symbol	52	-0.484	31.43	0.559	71.19	-0.231	40.88	-0.057	47.74	0.400	65.54
Boston Naming Test (30 odd)	25	-1.590	5.59	-0.843	19.96	-0.916	17.99	-1.251	10.54	-0.688	24.59

MMSE, Mini-Mental State Examination; WAIS, Wechsler Adult Intelligence Scale.

Severely impaired = < 1%, Moderately impaired = 1% to 1.99%, Mildly impaired = 2% to 8.99%, Low average = 9% to 24.99%, Average = 25% to 74.99%, High average = 75% to 90.99, Superior = 91% to 97.99%, Very superior = 98%<

The intended use of this calculator and any normative data used to inform assessment decisions is to provide objective data on an individual's performance relative to a group of people of similar backgrounds, but it does not replace the clinician's judgment, and, as with all statistical procedures, individual variability occurs. Clinical judgment should include a consideration of the objective test data, as well as the specific observations of the given individual being assessed. It is possible that the different percentiles obtained for different tests within the same domain are due to variability in the sensitivities across neuropsychological tests; it is also possible that individual variability of the examinee can produce this variability. As is the case in any statistically-derived estimate of normative performance, there is inherent error in our ability to predict performance at the individual level.

This study has several strengths and benefits that include measurement estimates representative of the NACC UDS and ADC/ADRC populations; utilization of methods and models that are straightforward, intuitive, and have been tested on a large sample of well-characterized subjects, and the provision of a simple and practically useful tool for UDS clinical researchers that builds on and complements available NACC-ADC/ADRC resources.

The study's results and approach also have several inherent limitations and caveats. First, as stated in Weintraub *et al*.'s original article [2], the majority of UDS participants are White, non-Hispanic, highly educated, and have few additional medical or psychiatric illnesses. Therefore, the application of this calculator may be best suited for individuals reflective of these characteristics. For example, if we were to compare our previous illustrative MMSE score to the MMSE normative information provided by Crum and colleagues [14], where the mean and standard deviation for a person with 12 years of education and 80 years of age is 25 ± 2.3, we would determine that the subject had a *z-score *of .87, fell in the 82^nd ^percentile, and has performed in the "high-average" range. Therefore, it is imperative that the context in which this calculator is used be one in which the subject shares similar demographics to those within the UDS sample.

The second potential limitation is the use of the RMSE in deriving *z-scores*. Although flexible in its application, the RMSE is calculated with the assumption that error variance is homoscedastic across changes in the predictor variable. While these regressions were performed in Weintraub *et al*. [2], this assumption may not hold in all instances. For example, as a cohort's age increases, the range of the cohort's scores on certain tests (for example, TMT B) also increases; this can weaken the assumption of homoscedasticity [15]. Therefore, *z-score *estimates for individuals who fall at the ends of the age range (that is, 60 or younger and 90 or older) may be relatively less informative. For example, if a 58-year-old were to truly perform in the mildly impaired range on the Trails B task compared to same-aged peers, this relatively poor performance may be masked because the overall range of scores would be overestimated due to the inclusion of the older cohort in estimating the RMSE, leading to a less severe interpretation. Conversely, a 95-year-old's seemingly low or impaired performance on TMT B may simply be an exaggeration due to an underestimation of her performance or due to a restricted estimation of the range as a result of including the younger cohort's scores in calculating the RMSE. Due to such potential for under- or over-estimation, scores for individuals falling at the tail ends of the age range (distributions) should be interpreted with caution. It is possible to develop other models that specifically model differences in variance across covariates (for example, age) to compare covariate-specific effects on estimated norms between models. However, in this paper we aimed to make use of the best available published UDS baseline model parameters (from Weintraub *et al*. [2]) to produce an estimated norms calculator of practical use to specific researchers (that is, UDS clinician researchers) as well as methods that are simple to implement and generalizable to other datasets; in doing so we chose practicality, utility, simplicity, and generalizability over *de novo *developing models with greater complexity but potentially improved accuracy. The latter can be explored in future studies by developing more complex models and leveraging additional UDS data.

Finally, these models were developed based on subjects who were deemed to be clinically cognitively-normal at their first UDS visit; yet, approximately 20% of the subjects had one or more neuropsychological test scores that were deemed impaired or lower than expected. This does not preclude that a substantial portion of these subjects, all of whom were initially deemed clinically cognitively-normal, when followed longitudinally, may ultimately manifest more clear deficits on subsequent UDS visits or meet the newly proposed Sperling and colleagues' NIA-AA research criteria [3] for pre-clinical AD, MCI or dementia. Inclusion of these subjects would be expected to produce even more conservative estimates of "abnormality". The calculation of such "robust norms" is important and is currently underway by Ferris and colleagues (S. Ferris, oral/written communication, October, 2010). Future directions include developing a UDS norms calculator that uses age-specific standard deviations instead of the RMSE to obtain standardized scores that are more sensitive to age-related changes in the range of scores across age cohorts.

## Conclusions

We provide an interactive, regression-based, normative score web-based calculator to serve as an additional resource for UDS clinical researchers to supplement other preclinical AD criteria [3]. This simple tool may be of practical use, especially to guide interpretation of individual performances that may appear to initially fall in borderline areas where thresholds between types of impairments are defined.

## Abbreviations

AD: Alzheimer's disease; ADC: Alzheimer's Disease Center; ADRC: Alzheimer's Disease Research Center; BNT: Boston Naming Test; CDR: Clinical Dementia Rating (Scale); FAQ: Functional Assessment Questionnaire; LMIIA: logical memory II, story A; MCI: mild cognitive impairment; MMSE: mini-mental state examination; MV: multivariate models; NACC: National Alzheimer Coordinating Center; NIA-AA: National Institute on Aging and Alzheimer's Association work-group; NPT: neuropsychological test; RMSE: root mean square error; SAS: Statistical Analysis Software; SPSS: Statistical Package for the Social Sciences; TMT: trail making test; UC: unconditional model; UDS: uniform data set; UV: univariate models; WAIS-R: Wechsler Adult Intelligence Scale-Revised; WMS-R: Wechsler Memory Scale-Revised.

## Competing interests

The authors declare that they have no competing interests.

## Authors' contributions

SS was involved in conceptualization of the study, conducted all statistical analysis, and was primarily responsible for drafting the manuscript. MM participated in conceptualization, interpretation of statistical analysis, and drafting and review of the manuscript. LS participated in interpretation of results and provided critical review of manuscript drafts. JS was involved in study conceptualization and provided critical review of the manuscript. JL participated in statistical analysis and provided critical review of the analysis and results sections of the manuscript. SW was involved in study conceptualization and provided critical review of the manuscript. AA was involved in study conceptualization, interpretation of data analysis, drafting of the manuscript, and provided critical review of all aspects of data analysis, interpretation, and manuscript preparation. All authors have read and approved the manuscript for publication.

## Supplementary Material

Additional file 1 **Normative Calculator for the Uniform Data Set (UDS)**. Neuropsychological Test Battery with illustrative example of demographics and test performance. Click here for file

Additional file 2 **Template for researchers to develop their own normative calculator using our methodology**. Click here for file
